# Border Security Fencing and Wildlife: The End of the Transboundary Paradigm in Eurasia?

**DOI:** 10.1371/journal.pbio.1002483

**Published:** 2016-06-22

**Authors:** John D. C. Linnell, Arie Trouwborst, Luigi Boitani, Petra Kaczensky, Djuro Huber, Slaven Reljic, Josip Kusak, Aleksandra Majic, Tomaz Skrbinsek, Hubert Potocnik, Matt W. Hayward, E. J. Milner-Gulland, Bayarbaatar Buuveibaatar, Kirk A. Olson, Lkhagvasuren Badamjav, Richard Bischof, Steffen Zuther, Urs Breitenmoser

**Affiliations:** 1 Norwegian Institute for Nature Research, Trondheim, Norway; 2 Tilburg University, Tilburg Law School, Department of European and International Law, Tilburg, The Netherlands; 3 Department of Biology and Biotechnology, University of Rome "La Sapienza," Rome, Italy; 4 Research Institute of Wildlife Ecology, University of Veterinary Medicine Vienna (Vetmeduni Vienna), Vienna, Austria; 5 Faculty of Veterinary Medicine, University of Zagreb, Zagreb, Croatia; 6 University of Ljubljana, Biotechnical Faculty, Department of Biology, Ljubljana, Slovenia; 7 Schools of Environment, Natural Resources and Geography, Bangor University, Bangor, Gwynedd, United Kingdom; 8 Biodiversity Institute Oxford, Department of Zoology, The Tinbergen Building, Oxford, United Kingdom; 9 Wildlife Conservation Society, Mongolia Program, Ulaanbaatar, Mongolia; 10 Institute of General and Experimental Biology, Mongolian Academy of Sciences, Ulaanbaatar, Mongolia; 11 Norwegian University of Life Sciences, Department of Ecology and Natural Resource Management, Ås, Norway; 12 Association for the Conservation of Biodiversity of Kazakhstan (ACBK), Astana, Kazakhstan; 13 KORA, Muri bei Bern, Switzerland

## Abstract

The ongoing refugee crisis in Europe has seen many countries rush to construct border security fencing to divert or control the flow of people. This follows a trend of border fence construction across Eurasia during the post-9/11 era. This development has gone largely unnoticed by conservation biologists during an era in which, ironically, transboundary cooperation has emerged as a conservation paradigm. These fences represent a major threat to wildlife because they can cause mortality, obstruct access to seasonally important resources, and reduce effective population size. We summarise the extent of the issue and propose concrete mitigation measures.

## Abstract 2

While cooperation across national boundaries has emerged as a key concept in wildlife conservation, this Perspective reveals that, at the same time, there has been a dramatic increase in the extent of security fencing along international borders, which has dramatic consequences for this approach.

## The Rise of Transboundary Conservation

Conserving biodiversity on an increasingly crowded planet will always involve a combination of applying ecological knowledge and skilful politics. The art of successful conservation lies in aligning the best available knowledge with the appropriate management actions and the current political and economic realities. Accordingly, conservationists must constantly adjust their strategies to the prevailing opportunities and constraints within a constantly shifting environment.

The period from the early 1980s through the dawn of the 21st century was marked by a fortuitous convergence of situations. Environmental awareness among the general population was high, the Cold War was ending, and a range of international legal instruments—e.g., Convention on International Trade in Endangered Species (CITES), Convention on Migratory Species (CMS), Bern Convention on European Wildlife Conservation, the European Union (EU)’s Birds and Habitats Directives, and Convention on Biological Diversity (CBD)—emerged to codify regional and global efforts to conserve biodiversity and guide society toward a more sustainable path.

One key element of this emerging effort was an increased awareness of the large scale at which ecological processes occur and the realization that achieving collective goals would require international cooperation. This led to a diversity of actions, including global efforts to reduce pollutants, prevent the ozone hole from expanding, and halt climate change. On a more regional level, there was an increase in international cooperation to conserve wildlife populations that roamed across international borders. This new focus gave rise to transboundary protected areas that benefited from the removal of many border fences that had obstructed wildlife movements for decades. The fall of the Iron Curtain in 1989 and the emergence of the European Green Belt conservation initiative [1] were both symbolic of a new borderless world and provided new opportunities to restore habitat connectivity on a continental scale. This was also a period of increased regional cooperation and a greater flow of people and goods (e.g., the development of the EU).

In part due to the harmonisation of legislation across borders and restored connectivity, Europe has witnessed a tremendous recovery of its large carnivore and herbivore populations in recent decades, most visibly demonstrated by the expansion of wolves (*Canis lupus*) to areas of western Europe from which they had been functionally absent for more than half a century [2]. The extensive spatial requirements of large carnivores made them obvious flagships for transboundary cooperation, and the Council of Europe's Bern Convention was a very early advocate for this approach, producing a long series of regional recommendations calling for cooperation. The EU later developed formal guidelines for transboundary cooperation within the frames of the Habitats Directive [3]. Although implementation has been slow at the political level, a high degree of administrative and research cooperation across borders exists [4], and the large carnivores have benefitted from their newfound access across the continent's increasingly invisible borders. The need for, and the benefits arising from, transboundary cooperation adjusted to wildlife populations rather than jurisdictional units has led to its emergence as the major conservation paradigm for large carnivores and much other wildlife in Europe, and is increasingly being applied elsewhere [5–7].

## Geopolitical Forces and the Return of Borders

**Fig 1:**
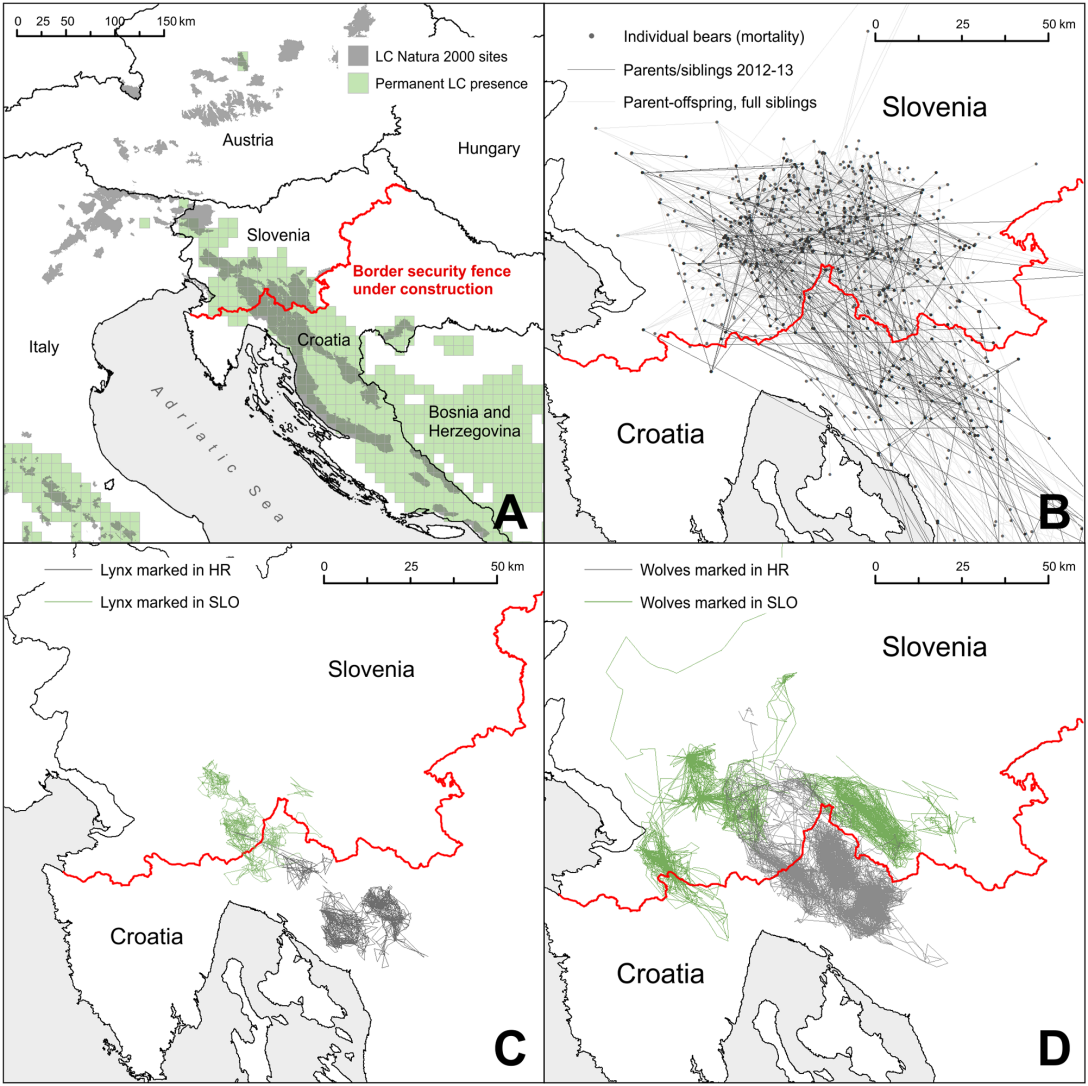
A: A border security fence being constructed along the border between Slovenia (SLO) and Croatia (HR) separates all three large carnivore (LC) species in Slovenia from the core population areas in the Dinaric Mountains, impacting their long-term viability, severing the Natura 2000 network, and decreasing the potential for natural recolonization of the Alps. B: The expected effect of the fence on brown bears. Points are individual bears, genetically sampled from bear mortalities between 2003 and 2013 (*N* = 1,414), which we genotyped using 20 polymorphic microsatellite loci. Lines showing full siblings or parent–offspring relations between individuals demonstrate that the border between both countries, where the fence is now being constructed, had, up until now, no effect on movement and gene flow in this bear population. The Trio ML method (Wang 2007) was used to estimate relatedness. Lines show *r* > 0.45. Darker lines are assignments only for bears sampled in 2012 and 2013 (*N* = 296). C,D: GPS telemetry tracks of lynx (C, *N* = 11) and wolves (D, *N* = 28) show that these animals had no problems crossing the border before construction of the fence, and even had home ranges that straddled the border.

Suddenly, in 2015, Europe received a massive-scale influx of refugees fleeing conflicts in Syria, Iraq, Afghanistan, and the Horn of Africa. The rapid erection of hundreds of kilometres of border security fences on both the external and internal borders of the EU was one of many responses to the perceived challenges associated with these refugees (Fig 1; Box 1). These fences were erected as emergency measures with no environmental impact assessments concerning their design or placement. Conservationists were quick to join those already protesting against these fences on humanitarian grounds, and images of red deer (*Cervus elaphus*) dying after becoming entangled in the coils of wire made media headlines in the region. The result has forced us to realise that the transboundary paradigm as we know it is gravely threatened.

**Box 1. Bears, Lynx, and Wolves in Slovenia and Croatia**

When Hungary closed its border to refugees in the summer of 2015, Slovenia became the main transit country for refugees on the way to Western Europe. The Slovenian government decided in November 2015 to construct a razor-wire security fence along large parts of the country’s 670 km border with Croatia. Such a fence likely has considerable unintended consequences for nature conservation. In the Dinaric Mountain range, 349 km of the fence will cut through some of the best-preserved natural areas of the region (Fig 1). Most of that area is covered by the Natura 2000 network and harbors many rare or endangered species, including three of Europe’s large carnivores: the brown bear (*Ursus arctos*), the gray wolf (*Canis lupus*), and the Eurasian lynx (*Lynx lynx*). The Dinaric Mountains contain one of the largest and most important brown bear and wolf meta-populations in Europe, stretching from Greece to the Alps. The lynx population straddling the Slovenia–Croatia border is currently threatened by its small size and high degree of inbreeding. All three species are considered a conservation priority in the EU and are listed in Annex II and Annex IV of the EU Habitats Directive (Directive 92/43/EEC). Large spatial requirements and low population densities make conservation of these species particularly challenging, and the current successes in their conservation rely largely on the ability of individuals to move between subpopulations. For these reasons, the Habitats Directive specifies that EU Member States must establish species-specific interconnected networks of protected areas. The security fence is likely to interrupt the existing connectivity.

The bear populations in both countries are large enough to persist in the short term. However, the fragmentation of the bear population implies that both countries will have to adapt their hunting/culling regimes to a situation in which there is much less scope for cross-boundary movements of individuals to buffer against local over-harvest. This will require much more caution in harvest when the former continuous population is managed as separate smaller units.

Wolves may face a more serious challenge. Out of 10 or 11 wolf packs currently present in Slovenia, five have their home ranges on both sides of the Slovenia–Croatia border. While wolves have shown an ability to cross different linear barriers, there are no guarantees that the wolves in Slovenia would remain connected with the core meta-population in the south. In isolation, they would face rapid inbreeding and vulnerability to demographic stochasticity, making viability of such a population fragment questionable. Wolves are also legally culled in Slovenia—a practice that may have been defendable before construction of the fence, but which will need to be reconsidered in the future. For the Dinaric lynx, the construction of the razor wire fence may just be the last push for the population to spiral down the extinction vortex.

While the fence remains in place, the conservation status of large carnivore populations in the Northern Dinaric Mountains should be reassessed, and management modified accordingly. The fence is in direct conflict with one of the main targets of the EU Biodiversity Strategy to 2020 (EBS) and the EU Green Infrastructure Strategy for achieving the ESB targets, aimed at restoring and maintaining habitat connectivity for a range of species. If the fence becomes a permanent feature it can undo decades of conservation and international collaboration efforts.

**Fig 2:**
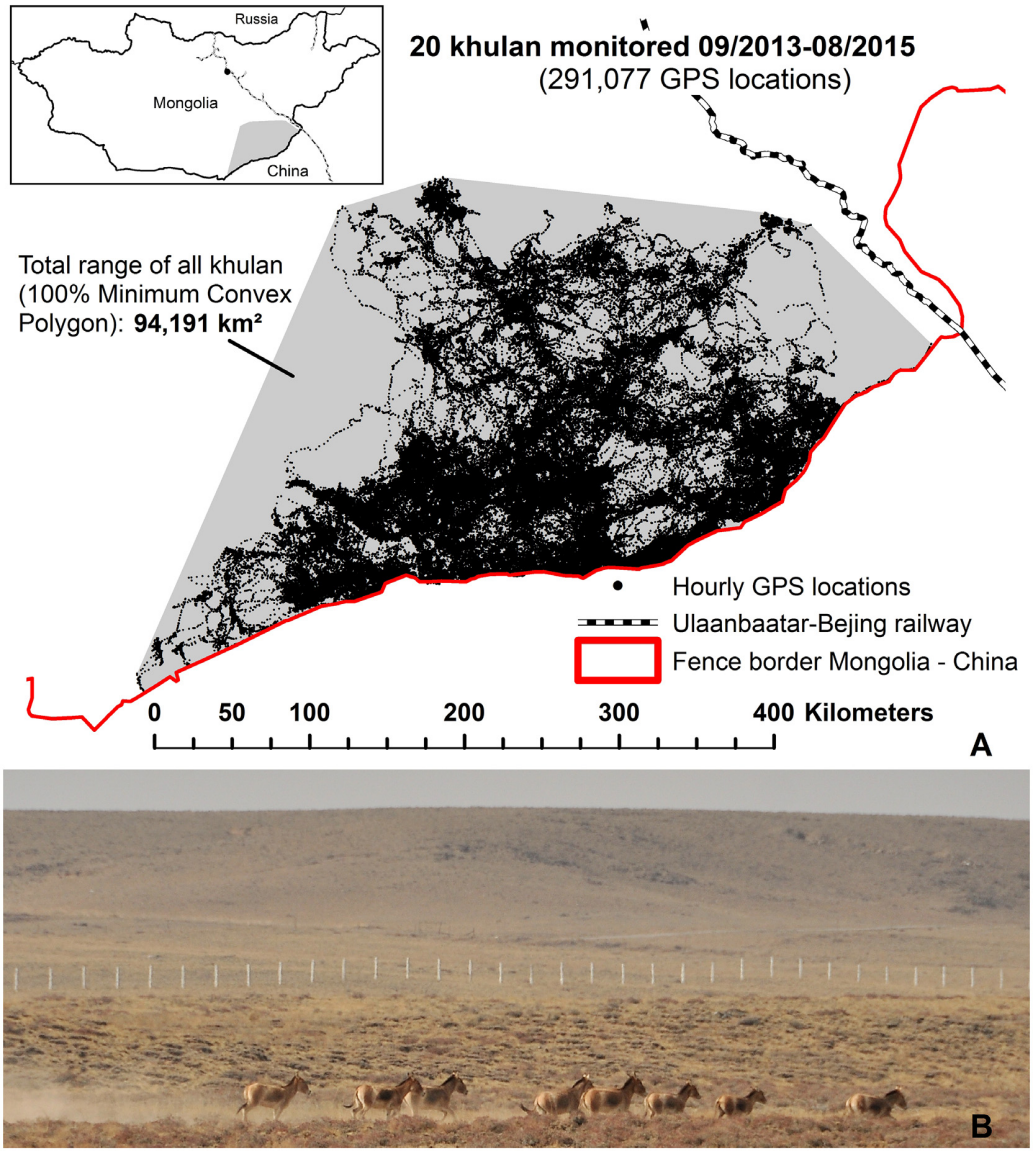
A: The security fence along the Mongolian–Chinese border constitutes an absolute barrier for movements of khulan and other large herbivores in the southeast Gobi. B: A group of khulan in the no-man’s land between the Mongolian (not seen) and Chinese border fence (background). *Photo*: *Petra Kaczensky*

Parallel processes have been ongoing further east in Eurasia (Fig 2; Table 1; Box 2). The collapse of the Soviet Union resulted in new political constellations, not only in Europe, but in the Caucasus and Central Asia. Newly independent countries emerged, facing dramatic socioeconomic and political changes. Recognising the opportunities and challenges for transboundary wildlife conservation in this region, the parties to the CMS, “concerned particularly with those species of wild animals that migrate across or outside national jurisdictional boundaries,” adopted a Central Asian Mammals Initiative (CAMI) in November 2014. In addition, what were formerly political alliances are being reborn as newly emerging economic alliances, as in the case of the Eurasian Customs Union, which consists of Belarus, Russia, Kazakhstan, Kyrgyzstan, and Armenia. On the one hand, many of the new countries in the region are CMS parties, and the importance of Central Asia as a hotspot for the conservation of large herbivore migrations is being increasingly recognized. On the other hand, in response to recent regional security concerns—for example, the conflicts in Afghanistan and ongoing tensions between neighbors [8]—border fences have been retained, re-established, reinforced, or newly erected, leading to increased mortality and fragmentation of wildlife.

**Table 1:** Summary of extent of border fencing across Europe, the Caucasus, and Central Asia, and the species of large mammals that this fencing can potentially have the largest impacts upon. Separation between primary and secondary impacts is based on our perception of their ability to cross fences. Full details of the specific borders are provided in the Supporting Information, but it is important to note that there is considerable uncertainty over the length of fencing on some borders.

Region	Number of		Estimated length of fencing (km)	Species affected	
	borders with fences	countries involved		primary	secondary
***Europe***					
EU–EU	5	5	350–450	Brown bear, red deer, roe deer, chamois	Eurasian lynx, wolf, wild boar
EU–non-EU	13	14	>2,250	Brown bear, red deer, roe deer, wild forest reindeer, moose, European bison	Eurasian lynx, wolf, wild boar
Non-EU–non-EU	3	3	>160	Brown bear, red deer, roe deer, moose	Eurasian lynx, wolf, wolverine, wild boar
***Caucasus***	10	11	>1,880	Brown bear, red deer, roe deer, Caucasian tur, chamois, mouflon	Eurasian lynx, wolf, leopard, wild boar, stripped hyena
***Central Asia***	25	13	>21,000	Asiatic wild ass, Mongolian gazelle, saiga, black-tailed gazelle, chinkara gazelle, urial, argali, markhor, Siberian ibex, bezoar ibex, wild camel, Asiatic cheetah, tiger, brown bear, Asiatic black bear, moose, Siberian roe deer, red deer, Siberian musk deer, Przewalski's horse	Leopard, snow leopard, Eurasian lynx, wolf, wild boar, stripped hyena, wolverine

**Box 2. Case Study of Khulan in Mongolia**

The 4,710-km Mongolian–Chinese border is fenced almost in its entirety. Asiatic wild ass (*Equus hemionus*, khulan in Mongolian) equipped with GPS collars in 2013 have demonstrated that the border fence presents an absolute barrier for khulan (Fig 2, [9]). The same has been observed for Mongolian gazelles (*Procapra gutturosa*) along the border fence with China and Russia [10,11]. While the fence restricts khulan movements, the associated 10-km limited entry security zone seems to have become a pasture refuge for khulan in the resource-poor winter season (e.g., in January 2014, 64% of all locations were concentrated in this area). A subsequent ground survey confirmed the presence of large herds of khulan along the border in late winter and additionally documented the presence of khulan in the no-man’s land between the Mongolian and Chinese fence lines. This has become possible because the Mongolian fence has fallen into disrepair in multiple places. The Chinese fence, on the other hand, has been newly constructed and acts as the actual barrier. Whilst a majority of the global khulan population still roams across Mongolia’s southeastern Gobi, their presence in the adjacent Chinese autonomous region of Inner Mongolia seems to be only sporadic, and illegal hunting appears to remain a major problem [12]. Given the uncertain population status of khulan in Inner Mongolia, increasing the fences’ permeability for khulan may not be advisable. Furthermore, recognizing the importance of the border area as a grazing refuge will enhance khulan conservation in Mongolia’s southeastern Gobi. In the Dzungarian Gobi in southwestern Mongolia, the situation is very different, as there are large populations of khulan on both sides of the fence. Furthermore, in the adjacent Chinese autonomous region of Xinjiang, firearms are tightly controlled, and illegal hunting does not seem to constitute a major problem. Thus, in the southwestern Gobi, connectivity of khulan and other far-ranging wildlife would greatly benefit from implementing wildlife crossing possibilities between two protected areas in Mongolia and a nature reserve in China [9,13].

Fencing serves many purposes, and both the positive and negative effects of various types of fencing (livestock fencing, veterinary cordon fencing, protected area perimeter fencing, road/railway fencing) on wildlife is well documented. However, the published literature on the impacts of international border security fencing on wildlife is minimal. Border security fencing is in a category of its own because of the extensive length, restricted access locations, and challenges associated with mitigating the fences’ effects on wildlife populations without compromising their intended security purposes. Accordingly, we sought to construct a Eurasian-wide overview (Europe and Central Asia) of the extent and implications of this form of fencing, based on our area of expertise and to align with our ongoing work with the science–policy interfaces of the Bern Convention, the Habitats Directive, and the CMS Central Asian Mammals Initiative. To do so, we surveyed the peer-reviewed and grey literature (mainly from the conservation biology and geopolitical disciplines), searched the online news media for relevant articles, photographs, and blogs, examined multiple images posted on Google Earth, and enquired among our colleagues working in many countries, as well as pooling our own field experience and observations from working in most of the affected countries. In addition, we performed an assessment, employing standard legal research methodology [14], of the compatibility of border fences with relevant international legal instruments in the field of wildlife conservation. This piece of the legal and policy puzzle has been largely overlooked, with legal scholarship hitherto focusing mainly on the implications of fences in terms of human rights and refugee law.

## Border Security Fencing and Wildlife

**Fig 3:**
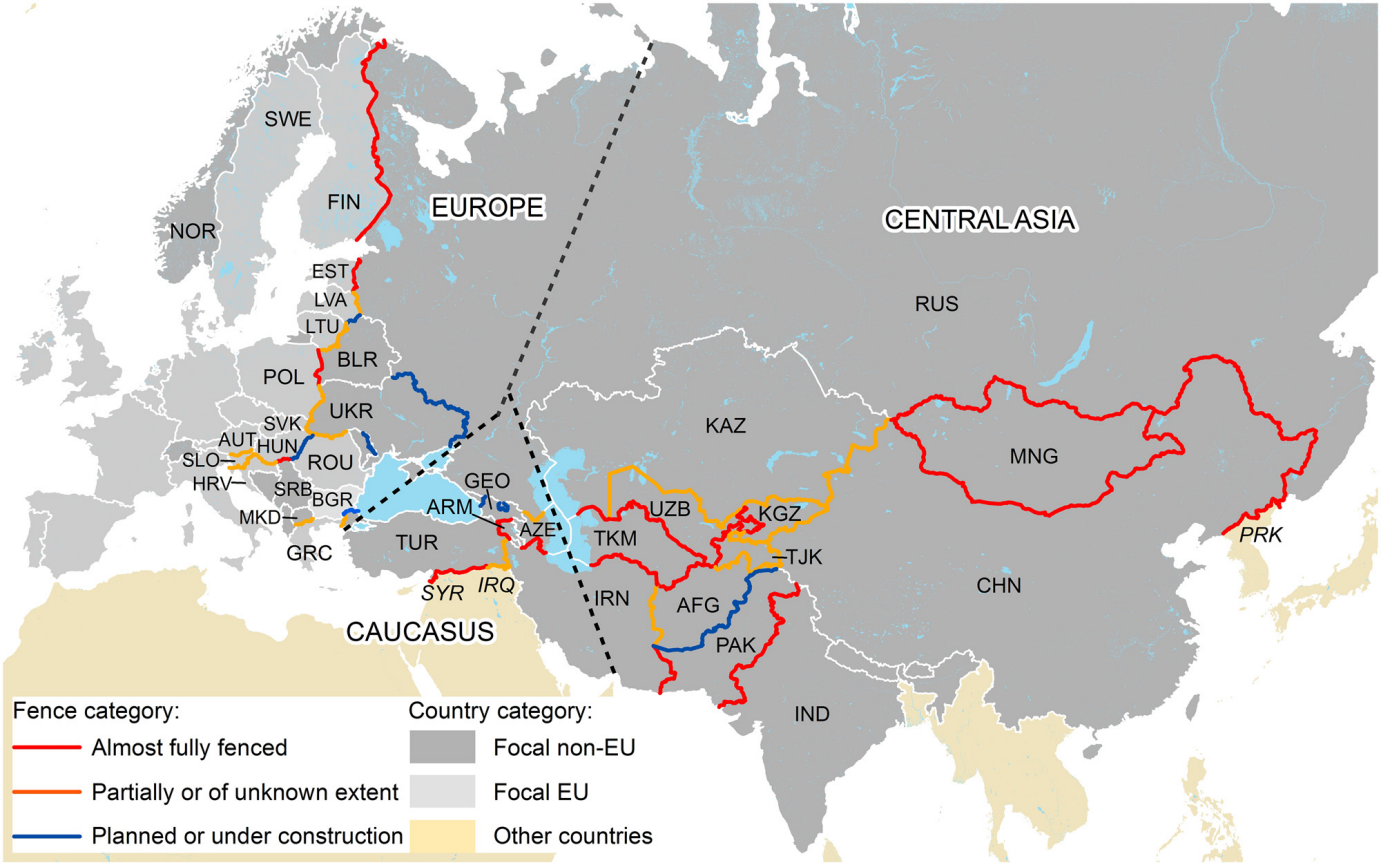
Extent of border security fencing along national borders in Europe and Central Asia.

Our first finding is that concrete information on this topic is very hard to find, especially concerning the exact location, length, and construction of the different fences (Fig 3; Table 1; S1 Appendix). This is not surprising considering that such fencing falls under national security considerations and is often located in border zones with restricted access. There are a number of overviews of the topic in the popular media and the academic geopolitical literature [15–17]. However, the sources of the material are unclear, and the same examples (e.g., the Mongolia–China, Turkmenistan–Iran, and Turkmenistan–Afghanistan border fences) are missing from all such overviews, reflecting the difficulties of collecting data and a resulting dependence on the same fragmented sources. The best documented studies came from outside our study area, namely, the United States–Mexico border [18] and those in the Middle East [16].

A second finding is that, even based on our fragmented and incomplete overview, there appears to be a massive amount of border fencing in the study area (in the order of 30,000 km). Several large countries have fenced in large parts, or all, of their national land borders (e.g., Russia, Turkmenistan). More unexpected is the observation that the extent of this fencing has been increasing during the 21st century, in part as a response to the post-9/11 security situation [19]. It is somewhat ironic that for the last 15 years, while conservation biologists have been largely promoting transboundary management and celebrating localised examples of fence removal, the global trend has been for an unprecedented increase in barriers preventing wildlife from moving across borders.

A third finding concerns the highly varied designs utilized for the border security fences. These details of design are crucial, as they will influence the extent to which a given fence functions as a barrier or source of mortality to different wildlife species. Unfortunately, a systematic overview of these details is lacking, making it impossible to conduct any form of spatially explicit analysis of the real fragmentation effect of these structures. There are likely to be very different effects (Table 1) of structures on different species, migratory large herbivores [9] and large carnivores being most affected. Only a few studies have analysed the impact that such barriers have (e.g., [20,21] for the Russia–Finland border, [9,11,22] and Box 2 for Mongolia, [23] for Kazakhstan, and [24] for the Polish–Belarussian border). Unfortunately, the latest generation of fencing being deployed is likely to constitute an even greater barrier than the older models.

Our fourth finding concerns the fences’ (il)legality. Generally speaking, international law does not forbid the construction of border fences by states on their own territories, unless such construction is at odds with specific obligations binding the state in question [25], such as human rights treaties or wildlife conservation treaties. Most of the major wildlife treaties at global and regional levels stress the need to avoid and remedy fragmentation and ensure adequate connectivity, whether in their binding provisions, in subsequent decisions adopted by their parties, or both [25,26]. We have identified several binding provisions which may, depending on the circumstances, be violated through the construction of border fences. Most notably, these include Articles 4, 5, and 6 of the United Nations Educational, Scientific, and Cultural Organization (UNESCO) World Heritage Convention, Articles II and III of the CMS, Articles 8 and 14 of the CBD, Articles 2 and 4 of the Bern Convention, and Articles 6 and 12 of the Habitats Directive. To illustrate, CMS Article III(4) commits contracting parties “to prevent, remove, compensate for, or minimize, as appropriate, the adverse effects of activities or obstacles that seriously impede or prevent the migration of the species” listed in Appendix I of the Convention. The latter lists several species potentially affected by border fences in our study area, including Bukhara red deer (*Cervus elaphus yarkandensis*), wild camel (*Camelus bactrianus*), Asiatic cheetah (*Acinonyx jubatus*), and snow leopard (*Uncia uncia*). Relevant non-binding instruments within the broader CMS framework include the aforementioned CAMI and two Memoranda of Understanding, for Bukhara deer and saiga antelope (*Saiga tatarica*), respectively.

Another key provision is Article 6 of the Habitats Directive, which lays down a stringent procedure to be followed by EU member states with regards to projects that may adversely affect species for which protected sites have been designated as part of the Natura 2000 network. Such a project may only go ahead if (i) a prior comprehensive assessment has conclusively proven that no significant adverse effects will occur, or if (ii) the project needs to be carried out anyway for “imperative reasons of overriding public interest,” alternative solutions have been proven absent, and compensatory measures are taken to ensure the overall coherence of the Natura 2000 network. In addition, for “priority species” such as wolf and brown bear (*Ursus arctos*), the opinion of the European Commission must be sought before proceeding, unless “public safety” is the reason for the project involved. Several Natura 2000 sites situated in affected border areas are home to (and designated for) wolves, brown bears, and Eurasian lynx (*Lynx lynx*) that are part of transboundary populations. Border fences fragmenting the animals’ habitat and impeding mobility and gene flow—the Slovenia–Croatia fences (Box 1) being a clear example—are evidently subject to the requirements of Article 6. Unfortunately, the conclusion appears warranted that this provision has been violated on more than one occasion through the hasty erection of border fences affecting Natura 2000 sites in various European countries.

## Recommendations for Mitigating Negative Impacts of Border Security Fencing

Based on the above findings, we have identified a set of recommendations that lie loosely along the framework of the mitigation hierarchy. Firstly, we recommend that conservation biologists increase their presence and profile in national and international debates surrounding border security fencing. Fences are expensive to build and maintain and impose significant transaction and opportunity costs on individuals and nations. It is essential that such decisions are not taken lightly, and they should be based on a transparent assessment of the full range of costs and the benefits that they will bring [16], related to other means to achieve the same goals, and generally be in conformity with applicable legislation. In cases in which fences are erected as "temporary or emergency" measures, it would be hoped that they be removed as soon as possible. While we do not expect the cost for wildlife to weigh as heavily as security, we believe that it is a significant issue that needs to be considered. A key element here is to raise awareness of the often key role played by border areas as refuges for wildlife.

Secondly, detailed planning to mitigate some of the undesired negative side effects of border fencing on wildlife is needed (Table 2). A wide range of high-tech monitoring methods are now available that would allow selected sections of a border to remain unfenced, while still providing security. The areas of greatest importance for wildlife are often remote and rugged, and there may be large gains to be made for wildlife with little compromise of security. More thoughtful fence alignment may also create opportunity to mitigate their effects. There are also examples of sections of border fences being temporarily removed to permit seasonal movements of migratory species [11,22]. Finally, it is important that wildlife-friendly fence designs that minimize the chance of entanglement and mortality are used. Such designs have been successfully retrofitted along border fencing between Kazakhstan and Uzbekistan on the Ustyurt Plateau to enable saiga antelope to pass between the two nations, and there may be a scope to develop similar structures for other species [22]. Animal tracking data and habitat suitability analyses supported by remote sensing can help guide fence construction and identify the best locations for mitigation measures [27]. The ongoing work with transboundary peace parks provides at least one institutional framework to focus mitigation actions into focal areas.

**Table 2:** The potential effects on wildlife of border security fencing and potential measures that can mitigate or compensate for their negative impacts. The importance of the different impacts will vary between species, depending on habitat, movement ecology, size, behaviour, and population density.

**Potential negative impact**	**Potential mitigation measures**
Mortality following entanglement	Fence design; avoid coils of concertina wire on the ground and certain structures involving parallel strands of barbed wire, especially close to the ground.
Mortality through electrocution	Fence design; ensure lowest electric wire allows small animals to pass underneath or is far enough from the main fence to allow them refuge from constant shocks.
Obstruction of small-scale/short-term movements and blocking of access to key resources	Careful design of fence routing in the landscape and provision of artificial resources (such as artificial water points).
Obstruction of seasonal migrations and dispersal	Wildlife crossing structures, careful design of fence construction, and carefully targeted (in space and time) openings combined with non-fencing border security infrastructure. Adjustment of harvesting plans and conservation actions to reflect greater population isolation.
Genetic fragmentation of populations	Translocation of individuals as a form of assisted dispersal.
Habitat loss and disturbance due to access roads and border security activities	Ensure that border security staff do not poach or harass wildlife.
**Potential positive impacts**	**Enabling requirements**
Prevents the smuggling of wildlife parts across borders	Requires that border crossing check points also enable effective searches for wildlife smuggling products.
Limited entry zones along international borders, including fenced no-man’s land, constitute refuge from human disturbance and grazing competition with livestock	Requires that border guards do not illegally kill wildlife and that crossing points for wildlife to access the area are available.

Thirdly, we need to realign our conservation paradigms with the political reality on the ground for large parts of Eurasia. The opportunities for transboundary cooperation in wildlife conservation are shrinking in many regions. When examining the geopolitical situation and the very real security challenges that some countries in Eurasia are facing at the moment, it seems likely that many of these fences are here to stay and that more are likely to appear, while existing fences are strengthened. This means that conservationists will have to recognise the potential impacts of these fences and adapt population management accordingly. In practice, the populations being managed will be smaller, unable to move as needed to reach seasonal habitats, and therefore, more vulnerable, thus requiring a greater degree of caution in management. It also implies that there may be a need to compensate for the impacts of habitat fragmentation by providing supplemental resources (such as water) to which access has been denied. In the long term, it may even be necessary to consider translocation of individuals as a form of assisted dispersal to maintain gene flow (Table 2).

Fourthly, we need to improve our knowledge and understanding about border fences and their effect on wildlife. While this may be a challenging task in many countries because of the desire for secrecy, it should still be possible to obtain coarse-scale data that facilitates conservation planning without compromising national security. Including border security personnel in wildlife monitoring is one critical step to raise awareness of wildlife needs, help assess the impact of border fences, and explore potential strategies for reducing the unintended impacts on wildlife. The United States National Military Fish and Wildlife Association (http://www.nmfwa.net/) offers many examples of wildlife conservation in military areas. Furthermore, the future development of such fences needs to be carefully monitored such that further constraints on animal movement can be identified rapidly. This is a suitable field for close cooperation between wildlife biologists and researchers within the field of geopolitics.

Finally, it must be recognised that, in some cases, border fencing may unintentionally actually help conservation by preventing animals from roaming into countries with low degrees of law enforcement (population sinks), by creating well-guarded spaces where human impact is minimal and that, in certain circumstances, constitute wildlife refugia, and by preventing the spread of wildlife diseases (Table 2). Furthermore, wildlife conservation may indirectly benefit from the circumstances of improved national security in countries where border fencing serves its intended functions and creates a secure political environment conducive to the effective institutions that are essential to reach social and conservation objectives.

## The End of the Transboundary Paradigm?

In summary, it would appear that geopolitical change has occurred at such a pace that conservation biologists have been left behind so that, while the transboundary paradigm has been advocated, it has been rendered less practical in many areas by the expansion and upgrading of border fences. While there is still a very large scope for wildlife to roam across borders in much of Western and Central Europe, the opportunities in Eastern Europe and Central Asia are decreasing. Hope lies in the ability to adopt a nuanced and context-specific view of both the motivations to build fences and the solutions to their side effects. Different border security fences are built for diverse reasons, including a response to the threat of military invasion, halting the movement of terrorists and insurgents, drug smugglers, and refugees, and as symbolic markers of territorial integrity or territorial claims. As such, the extent to which fences of different types (and with different effects on wildlife) are needed varies with context. In the context of the EU, it can only be hoped that the recently constructed fences, at least those within the EU, will be removed as soon as other measures are put in place to tackle the current refugee crisis. This constitutes a relatively low security threat that can probably be addressed in many other ways that have fewer unwanted side effects, at least compared to the threats facing other countries in Eurasia. The Baltic States are currently considering the construction of fences along their borders with Russia and Belarus, and it would be hoped that these would be subject to a full environmental impact assessment and designed in a way to take the needs of wildlife into account. Although Central Asia is one of the most heavily fenced regions in the world, the region benefits from relative political stability (with the exception of Afghanistan), which should open opportunities for the incorporation of wildlife crossing features into border security fencing [20]. However, we also recognise that the dominant political direction at present is for a clear strengthening of the external borders of both the EU and the Eurasian Economic Union, implying that the best scope for rapid action lies within these bodies, rather than on their external borders. All these issues force us to realize that the state institutions responsible for border security are one of the major stakeholders with which wildlife conservationists need to engage. As in so many other cases, this underlines the need for building communication between science and policy arenas and for cross-sectorial cooperation between different government agencies.

## Supporting Information

S1 Appendix Reported lengths of border security fences in Europe and Central Asia as of 2015–2016. Data is collected from many secondary sources, and there is a high degree of discrepancy between different sources. The situation is also highly dynamic, with new fences being constructed or reinforced. The most credible figure for present status is highlighted in bold. All information should therefore be treated with caution.
(DOCX)

## Abbreviations

CAMI: Central Asian Mammals Initiative
CBD: Convention on Biological Diversity
CITES: Convention on International Trade in Endangered Species
CMS: Convention on Migratory Species
EBS: EU Biodiversity Strategy to 2020
EU: European Union
UNESCO: United Nations Educational, Scientific, and Cultural Organization
