# Supplementary material for: Border Security Fencing and Wildlife: The End of the Transboundary Paradigm in Eurasia?
Source: PLoS Biol. 2016 Jun 22;14(6):e1002483. doi: 10.1371/journal.pbio.1002483 (PMC4917236; doi:10.1371/journal.pbio.1002483)
Supplement: S1 Appendix — Data is collected from many secondary sources, and there is a high degree of discrepancy between different sources. The situation is also highly dynamic, with new fences being constructed or reinforced. The most credible figure for present status is highlighted in bold. All information should therefore be treated with caution. (DOCX) [file pbio.1002483.s001.docx]

**Supporting Information Appendix 1**

Reported lengths of border security fences in Europe and Central Asia as of 2015-2016. Data is collected from many secondary sources and there is a high degree of discrepancy between different sources. The situation is also highly dynamic with new fences being constructed or reinforced. The most credible figure for present status is highlighted in bold. All information should therefore be treated with caution.

| **Border** | **Length of border_1_** | **Length of fence** | **Fencing status** |
| --- | --- | --- | --- |
| **Europe** |  |  |  |
| Russia – Norway | 191 | 195_4_ | 2-3 m barbed wire fence_4_ |
| Russia – Finland | 1309 | All_5_ | 2-3 m barbed wire fence_5_ |
| Russia – Estonia | 324 | 70_4_ /106_3_ | Proposed_3,4_ on Estonian side. Russia has a 2 m barbed wire fence on their side_5_ |
| Russia – Latvia | 332 | 90_4,5_ | Proposed _4_ |
| Belarus – Latvia | 161 |  | Proposed _4_ |
| Belarus – Lithuania | 640 | 65_5_ | 2-3 m wire mesh topped with roll of concertina wire_4_ |
| Belarus – Poland | 418 | 418_5,6_ | 2.5 m barbed wire fence_5_ |
| Ukraine – Poland | 535 | Partial_4,5_ | 2-3 m barbed wire fence, not continuous and parts in poor repair_5_ |
| Ukraine – Slovakia | 97 | Partial_4,5_ | 2-3 m barbed wire fence, not continuous and parts in poor repair_5_ |
| Ukraine – Hungary | 128 | Partial_4,5_ | 2-3 m barbed wire fence, not continuous and parts in poor repair_5_ |
| Ukraine – Romania | 601 | Partial_4,5_ | Unknown |
| Ukraine – Russia | 1944 | **71**_4_ /2000_3_ | Under construction_4_ |
| Ukraine – Transnistria |  |  | Proposed_4_ |
| Hungary - Romania | 424 |  | Proposed_4_ |
| Hungary – Serbia | 164 | 175_3,4_ | 3.5-4 m wire mesh with coils of concertina wire at base and on top where possible, otherwise 1.5-2 m high fence of three coils of concertina wire stacked vertically |
| Hungary – Croatia | 348 | 41_3_ / 120_4_ | 3.5-4 m wire mesh with coils of concertina wire at base and on top where possible, otherwise 1.5-2 m high fence of three coils of concertina wire stacked vertically |
| Slovenia – Croatia | 600 | 136_4_ | Multiple coils of concertina wire stacked vertically |
| Austria – Slovenia | 299 | 3.7 km_4_ | Multiple coils of concertina wire stacked vertically and wire mesh |
| Greece – Macedonia | 234 | A few km_4,5_ | 3-4 m wire mesh, fronted and topped with concertina wire |
| Greece – Turkey | 192 | 10_1_ / **12**_4,5_ | 3-4 m wire mesh, fronted and topped with concertina wire |
| Bulgaria – Turkey | 233 | **50**_4_/160_4_/240_4,5_ | Under construction. 3 m wire mesh, fronted and topped with concertina wire |
|  |  |  |  |
| **Central Asia** |  |  |  |
| Turkmenistan – Iran | 1148 | Almost all | 3-4 m barbed wire, electric fence, sensors_4_ |
| Turkmenistan – Afghanistan | 804 | Almost all | 3-4 m barbed wire, electric fence, sensors, potentially more than one fence line _4_ |
| Turkmenistan – Uzbekistan | 1793 | 1619_12_ / 1700_3_ | 3-4 m barbed wire fence_4_ |
| Turkmenistan - Kazakhstan | 413 | 378_12_ | 3.5m high barbed wire fencing_4_ |
| Kazakhstan – Uzbekistan | 2330 | 45_3,4,12_ / **615**_9_ / Plan to fence all border_4_ | 1.7 m high wire fencing, 8 strands and 2 diagonals. |
| Kazakhstan - China | 1765 | 1533_2_ | Unknown |
| Kazakhstan – Kyrgyzstan | 1212 | Partial | 3 m high rolls of wire_4_ |
| Kazakhstan – Russia | 7513 | Partial | Unknown |
| Uzbekistan – Tajikistan | 1312 | 1199_12_ | Potentially mined_4_ |
| Uzbekistan - Kyrgyzstan | 1314 | 869_12_ / 890_3_ / 1099_2_ | Under construction _10_ |
| Uzbekistan - Afghanistan | 144 | 209_3,12_ | Electric fences, mines_7_ |
| Kyrgyzstan – Tajikistan | 984 | Partial | Unknown |
| Kyrgyzstan – China | 1063 | Unknown | Unknown |
| Tadjikistan – China | 477 | Unknown | 2-3 m barbed wire fence_4_. Old Soviet fence and new fence on Chinese side is uc_4_ |
| Tajikistan - Afghanistan | 1357 | Unknown | 2-3 m barbed wire fence_4_ |
| Iran – Afghanistan | 921 | 140_7_ / 700_12_ | 800 km of canals, 39 km of concrete walls, 140 km of barbed wire_7_ |
| Iran - Pakistan | 959 | 700_3,12_/ 909_2_ | Under construction _3_ At least part in concrete_4_ |
| Pakistan – Afghanistan | 2670 | 1500_12_ / 2400_12_,_3_ | Proposed _3_ / Under construction _8_ |
| Pakistan – India | 2440 | **1900**_10_ / 2172_2_ | 3.5 m, electric fence, not contiguous in rugged or swampy areas_10_ |
| Pakistan – India (Kashmir) | 750 | 550_12_,_3_ | Electric fence_4_. 3.5 m chain link plus coils. Lighting_10_ |
| Mongolia - Russia | 3543_14_ | Almost all of land border in most regions, c. 2863_11,13_ | Barbed wire fence_11,12_ |
| Mongolia – China | 4710_14_ | Almost all of land border, c 4489_5,13_ | Barbed wire fence |
| China - Russia | 4179 | Almost all | Wire fence |
| China – North Korea | 1352 | 1416_3,12_ | Increasing length of electrified wire_4_ |
| Russia – North Korea | 18 | 19_2_ | Unknown |
|  |  |  |  |
| **Turkey and Caucasus** |  |  |  |
| Turkey – Iran | 534 | Partially fenced_4_,_5_ | Barbed wire fence and / or concertina wire_5_ |
| Turkey – Armenia | 311 | 267_2,4,5_ | Barbed wire fence and / or concertina wire along almost all border. Some minefields_5_ |
| Turkey – Azerbaijan | 17 | 11_5_ | Barbed wire_5_ |
| Turkey – Syria | 899 | >800_12,4_ | Barbed wire fence and / or concertina wire along almost all border, currently being upgraded with 150km of wall, 365 km of ditches plus sensors and floodlights in sections_4_ |
| Turkey - Iraq | 367 | No fence_5_ | Water filled trenches are under construction. Some minefields_5_ |
| Georgia – Abkhazia | unknown | >40_4_ | Under construction_4_ |
| Georgia – South Ossetia | c. 400 | >27_4_ | Concertina wire, under construction_4_ |
| Armenia - Iran | 44 | Fenced, , but with some gaps in the planned peace park area (Arevik NP in Armenia and Dizmar PA in Iran)_5_ | Barbed wire_5_ |
| Azerbaijan – Russia | 338 | At least partially fenced in lowlands_5_ |  |
| Azerbaijan - Iran | 689 | Fully fenced apart from section adjacent to Nagorno Karabakh_5_ | Barbed wire_5_ |

**References**

1. Data obtained from CIA World Fact Book (online) without prejudice towards disputed borders or territory status

2. Rosière S, Jones R. Teichopolitics: Re-considering Globalisation Through the Role of Walls and Fences. Geopolitics. 2012;17:217-34

3. Wikipedia <https://en.wikipedia.org/wiki/Border_barrier>

4. Search of online news media in period January - April 2016

5. Personal communications and author observations

6. Kowalczyk R, Schmidt K, Jedrzejewski W. Do fences or humans inhibit the movements of large mammals in Bialowieza Primeval Forest? In: Somers MJ, Hayward MW, editors. Fencing for conservation: restrictions of evolutionary potential or riposte to threatening processes?2012. p. 235-43

7. Gavrilis G, Ivanov V, Laruelle M, Long A, Mansfield D, Safranchuk I, et al. Afghan narcotrafficking: The state of Afghanistan’s borders joint U.S.-Russia Working Group on Afghan Narcotrafficking. New York: East West Institute; 2015

8. Saleem Mazhar M, Goraya NS. Border issue between Pakistan & Afghanistan. South Asian Studies. 2009;24(2):204-20

9. Olson KA. Saiga Crossing Options: Guidelines and Recommendations to Mitigate Barrier Effects of Border Fencing and Railroad Corridors on Saiga Antelope in Kazakhstan.: Smithsonian Conservation Biology Institute; 2014

10. Biger G. Walls, fences and international borders. Studia z Geografii Politycznej i Historycznej. 2013;2:87-108

11. Chimeddorj B, Buuveibaatar B, Onon Y, Munkhtogtokh O, Reading RP. Identifying Potential Conservation Corridors Along the Mongolia-Russia Border Using Resource Selection Functions: A Case Study on Argali Sheep. Mongolian J Biol Sci. 2013;11(1-2):45-53

12. Jellissen SM, Gottheil FM. On the utility of security fences along international borders. Defense & Security Analysis. 2013;29(4):266-79

13. Olson KA, Mueller T, Leimgruber P, Nicolson C, Fuller TK, Bolortsetseg S, et al. Fences Impede Long-distance Mongolian Gazelle (Procapra gutturosa) Movements in Drought-stricken Landscapes. Mongolian J Biol Sci. 2009;7(1-2):45-50

14. Border Protection Authority of Mongolia <http://bpo.gov.mn/en/frontier/103/1206270002/detail>
